# An Exploratory Study on the Rapid Detection of Volatile Organic Compounds in Gardenia Fruit Using the Heracles NEO Ultra-Fast Gas Phase Electronic Nose

**DOI:** 10.3390/metabo14080445

**Published:** 2024-08-11

**Authors:** Wenjing Cai, Wei Zhou, Jiayao Liu, Jing Wang, Ding Kuang, Jian Wang, Qing Long, Dan Huang

**Affiliations:** 1 The First Hospital of Hunan University of Chinese Medicine, Hunan University of Chinese Medicine, Changsha 410007, China; caiwenjing915@hnucm.edu.cn; 2 State Key Laboratory of Chinese Medicine Powder and Medicine Innovation in Hunan (Incubation), Science and Technology Innovation Center, Hunan University of Chinese Medicine, Changsha 410208, China; zhouwei@stu.hnucm.edu.cn (W.Z.); ljy@stu.hnucm.edu.cn (J.L.); 3 Hunan Gardenia Industrial Technology Research Center, Yueyang 414100, China; wangbuyu2@htbn.com.cn (J.W.); kuangding@htbn.com.cn (D.K.); wangyun88@htbn.com.cn (J.W.); longqing_csu@csu.edu.cn (Q.L.)

**Keywords:** gardenia fruit, Heracles NEO ultra-fast gas phase electronic nose, volatile organic compounds, difference markers, hexanal

## Abstract

Gardenia fruit is a popular functional food and raw material for natural pigments. It comes from a wide range of sources, and different products sharing the same name are very common. Volatile organic compounds (VOCs) are important factors that affect the flavor and quality of gardenia fruit. This study used the Heracles NEO ultra-fast gas phase electronic nose with advanced odor analysis performance and high sensitivity to analyze six batches of gardenia fruit from different sources. This study analyzed the VOCs to find a way to quickly identify gardenia fruit. The results show that this method can accurately distinguish the odor characteristics of various gardenia fruit samples. The VOCs in gardenia fruit are mainly organic acid esters, ketones, and aldehyde compounds. By combining principal component analysis (PCA) and discriminant factor analysis (DFA), this study found that the hexanal content varied the most in different gardenia fruit samples. The VOCs allowed for the fruit samples to be grouped into two main categories. One fruit sample was quite different from the fruits of other origins. The results provide theoretical support for feasibility of rapid identification and quality control of gardenia fruit and related products in the future.

## 1. Introduction

Gardenia fruit is a popular functional food with a history of thousands of years of consumption. Common gardenia fruit-related foods include gardenia fruit tea, gardenia fruit candy, and gardenia fruit beverages. Botanically, gardenia fruit is the dried ripe fruit of *Gardenia jasminoides* Ellis (Fam Rubiaceae). It is harvested from September to November when it turns reddish-yellow, removed from the fruit stalk, and then dried. In traditional Chinese medicine, it is usually used to purge fire to relieve vexation, clear heat and drain dampness, cool the blood, and remove toxins; in topical applications, it disperses swelling and relieves pain. It is mainly used for febrile diseases causing vexation, dampness–heat jaundice, stranguria with difficult and painful urination, blood heat with hematemesis, red painful eye swelling, fire toxin sores, and ulcers; its topical applications include sprains and contusions [1].

As it is an important food with great development potential, an increasing number of researchers have focused on its pharmacological activity, chemical composition, related mechanisms of action, and safety. Modern research shows that gardenia fruit has a wide range of pharmacological activities, including liver protection and choleretic activity, anti-inflammatory activity, antioxidant activity, and as a treatment of central nervous system diseases (such as Alzheimer’s, Parkinson’s, and insomnia) [2,3,4,5]. Gardenia fruit mainly contains iridoid ether terpenoid glycosides, organic acid esters, and crocin. To date, the development and application of the main chemical components of gardenia fruit have involved medicine, food additives, dyes, cultivations of ornamental plants, and preservatives [6].

Volatile organic compounds (VOCs) are an important basis for judging the freshness and flavor of food [7]. Gardenia fruits from different sources produce different VOC profiles due to different growth environments and harvesting conditions. There are certain differences, such as the surface of Zhangshu gardenia being reddish brown or yellow brown, with a slightly acidic and bitter odor, or the surface of Jinxi Gardenia being dark red or reddish yellow, with a light and slightly sour taste. The content of effective ingredients in different gardenia fruits also varies, which directly affects the quality and clinical efficacy of the medicinal materials. However, in the market, there may be instances of mixing different types of gardenias or using inferior ones as substitutes. A comparative analysis of gardenia fruit VOCs from different sources can help to select gardenia fruit products that better meet flavor conditions according to the application requirements of gardenia fruit as a food. Further analysis of the content of specific active ingredients in gardenia fruit is conducive to selecting gardenia fruit with higher medicinal value as medicinal materials, providing new strategies for enterprises to control production process quality. At present, the quality control of gardenia fruit as medicinal materials is mostly focused on determining the content of a single component. For example, the 2020 edition of the *Chinese Pharmacopoeia* uses the content of geniposide as an evaluation indicator [1], which cannot fully reflect the intrinsic quality or the differences in gardenia fruit from different origins.

Flavor can directly reflect the intrinsic essence of food. It is the connection point between the external quality performance of food and its internal material basis [8]. The flavor of food is mainly composed of VOCs and non-volatile components. VOCs comprise alcohols, ketones, esters, hydrocarbons, and heterocyclic compounds, while non-volatile components in the fruit mainly contribute to umami, sweetness, and sourness [9].

In the field of food, quality testing is predominantly performed using chemical analysis techniques, such as liquid chromatography and gas chromatography–mass spectrometry. These methods are time-consuming, expensive, and require strict technical skills. There are also newer technologies that can distinguish different samples, such as traditional sensory testing, near-infrared spectroscopy (NIR) [10], gas chromatography–ion mobility spectrometry (GC-IMS) [11], and electronic sensors.

The main methods currently used for VOC detection are GC-MS [12,13], metal oxide sensors [14], optical sensing (absorption and fluorescence spectroscopy) [15], acoustic wave sensing, and near-infrared spectroscopy [10], and electrochemical sensing, e.g., using an electronic nose [13,16].

An electronic nose is a device designed to simulate the human olfactory system. It detects odors through a specific sensor array and uses a pattern recognition system to analyze complex odors [16]. The Heracles NEO ultra-fast gas phase electronic nose is based on dual fast gas chromatography technology and is designed for odor and aroma analysis. The Heracles NEO ultra-fast gas phase electronic nose achieves the separation and identification of VOCs based on the separation principle of gas chromatography, and it reflects the overall information of VOCs in a sample through an odor fingerprint spectrum [17]. It is equipped with dual chromatographic columns of weak polarity and medium polarity and dual FID detectors, which can separate more compound signals. Screened chromatographic peaks are used as sensors to conduct principal component analysis and discriminant factor analysis through chemometrics, establish various models to implement discriminant analysis of odor substances [18], and the Heracles NEO ultra-fast gas phase electronic nose has the advantages of high sensitivity, rapid analysis, high throughput, real-time detection, powerful data processing and analysis capabilities, and high stability. Heracles NEO ultra-fast gas phase electronic nose technology does not only detect qualitative or quantitative results of one or several components in a sample, but also comprehensively and quickly gives overall information on the VOCs in the sample [19,20]. Compared with GC-MS and GC-IMS, the advantage is that it not only conducts qualitative and quantitative analyses of samples, but also has aroma sensing technology and an olfactory identification threshold for specific compounds, which can provide sensory descriptions of the flavor of a sample. To date, it has been used in food, medicine, cosmetics, and other fields [21,22,23].

At present, there are many studies on the nutritional value and chemical composition of gardenia fruit, but research on the VOC profiles of gardenia fruit from different sources is relatively weak. The VOC profile affects the quality of gardenia fruit. This study is based on the Heracles NEO ultra-fast gas phase electronic nose combined with the AroChemBase database (2021 version, Alpha MOS Corporation, Toulouse, France). Principal component analysis (PCA) and discriminant factor analysis (DFA) models were used to compare differences in the volatile components of six batches of gardenia fruit from different sources. Additionally, this study provided sensory descriptions for the flavor characteristics of each gardenia product, offering technical support for the rapid identification and quality control of gardenia fruit and related products.

## 2. Materials and Methods

### 2.1. Materials

Six batches of gardenia fruit (from Tanghe County, Anyang, China; Zhangshu, Yichun, China; Jinxi County, Fuzhou, China; Qu Yuan Management District, Yueyang, China; Ningxiang, Changsha, China; and Zhangshu, Yichun, China) were selected as samples and named TZZ, ZSZZ, JXZZ, QYZZ, NXZZ, and DZZ, respectively.

### 2.2. Sample Preparation

Six batches of gardenia fruit samples from different origins were crushed in turn, passed through a No. 3 sieve, and sealed and refrigerated for later use. For electronic nose detection, 2.0 g of gardenia fruit sample was first weighed into a 20 mL headspace bottle dedicated to the electronic nose and then sealed with a PTFE septum. To avoid accidental errors and ensure test accuracy, this study set up 5 parallel samples for each sample, and finally placed the prepared samples on the automatic sampler device for further analysis.

### 2.3. Heracles NEO Ultra-Fast Gas Phase Electronic Nose Analysis Conditions

By optimizing the detection parameters of a previous study, the detection conditions for gardenia fruit using the Heracles Neo were determined as follows: sample bottle, 20 mL; sample quantity, 2 g; incubation temperature, 70 °C; incubation time, 20 min; initial trap temperature, 40 °C; final temperature of the trap, 240 °C; flow rate of the trap, 0 mL·min^−1^; capture duration, 42 s; inlet temperature, 200 °C; injection volume, 5000 μL; injection speed, 250 μL/s; injection duration, 35 s. The initial column temperature was 40 °C; the column temperature program heating mode was 0.5 °C/s to 150 °C and 3 °C/s to 250 °C; the acquisition time was 290 s; the detector temperature was 270 °C; and the fID gain was 12. A normal alkane standard solution (nC6–nC16) was used for calibration, the retention time was converted into a retention index, and then the compounds were qualitatively analyzed using the AroChemBase database (2021 version, Alpha MOS Corporation, Toulouse, France).

### 2.4. Statistical Analysis

The experimental data were analyzed by using principal component analysis (PCA) and discriminant factor analysis (DFA) utilizing AlphaSoft 17.0 (Alpha MOS Corporation, Toulouse, France). Origin Pro 2023 software (OriginLab Corporation, Northampton, MA, USA) was used to draw the bar graphs.

## 3. Results

### 3.1. Gas Chromatogram Analysis

**Figure 1:**
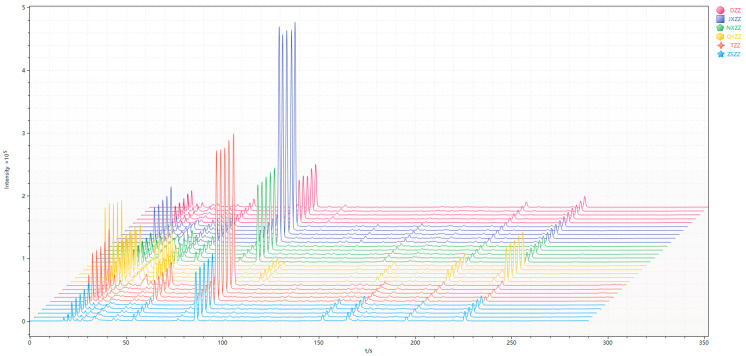
MXT-5 gas chromatogram overlay diagram.

**Figure 2:**
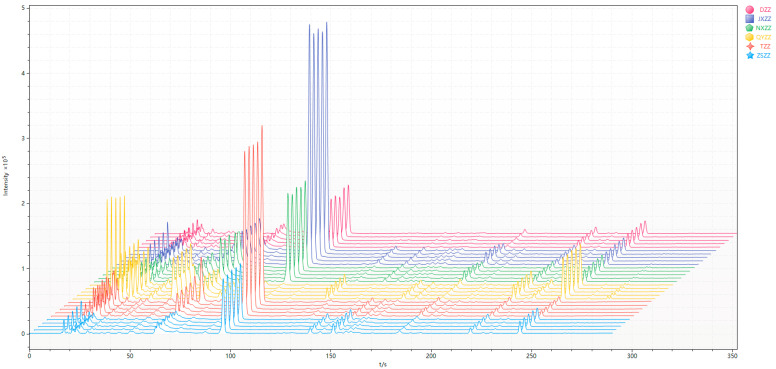
MXT-1701 gas chromatogram overlay diagram.

There are two ionization detectors in the Heracles NEO ultra-fast gas phase electronic nose, namely, the MXT-5 low-polarity chromatographic column and the MXT-1701 medium-polarity chromatographic column. In order to compare the differences between samples more accurately, this study used two ionization detectors. The detection results on the chromatographic column were analyzed, and the original gas chromatograms of all of the gardenia fruit samples were superimposed; the results obtained are shown in Figure 1 and Figure 2. The different colors in the picture represent different samples. It is evident from the gas chromatography overlay diagram that the detection results of the two chromatographic columns are generally similar. There are differences in the retention times and peak areas of the six gardenia fruit samples. As seen from the spectrum, the chromatographic peak of the QYZZ sample is generally higher between 0 and 50s, and there is a characteristic peak near 100s. After analyzing this peak, it can be observed that the 100s chromatographic peak of the JXZZ sample is higher than of the other five species. The color peak heights between 100s and 300s are relatively low for the gardenia fruit sample. Among them, the peak heights of the QYZZ sample and the ZSZZ sample are significantly different and are higher than the other samples at different peak times. Analyzing the original spectra, it is observed that the difference between the six gardenia fruit samples is mainly reflected in the change in peak height, that is, the difference in the number of VOCs. In order to further verify the differences between sample groups, this study first used PCA statistics to reveal the differences in VOCs between the sample groups, determined the differential chromatographic peaks, and then qualitatively analyzed the specific chromatographic peaks through database retrieval, to accurately and effectively reveal the differences in VOCs between the sample groups.

### 3.2. Principal Component Analysis (PCA)

PCA is a data dimensionality reduction analysis method. Through dimensional reduction processing and linear transformation of the original sample data, a two-dimensional PCA score chart is obtained, and the distance between sample points on the chart visually displays sample differences. Closer distances indicate smaller sample differences, while farther distances indicate greater differences.

**Figure 3:**
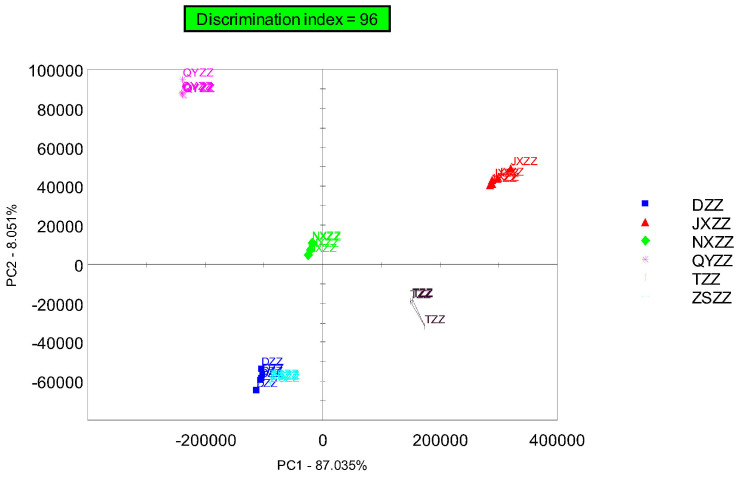
Principal component analysis of gardenia fruit samples.

PCA processing was performed on the data of six batches of gardenia fruit samples, and a principal component analysis chart was obtained, as shown in Figure 3. 

The horizontal and vertical coordinates, respectively, represent the contribution rates of PC1 and PC2 obtained in the PCA. The PCA model shows that the contribution rate of PC1 is 87.035%, the contribution rate of PC2 is 8.051%, and the cumulative contribution rate of PC1 and PC2 reaches 95.086%, which better reflects the sample actual situation. The identification index on the electronic nose principal component analysis chart of the sample reached 96%, which shows that some differences in VOCs exist among the six batches of gardenia fruit samples. Heracles NEO ultra-fast electronic nose technology can effectively distinguish gardenia fruit samples. As seen from the figure, ZSZZ and DZZ have the closest position distribution, and the overall odor difference between the two groups of samples is small. QYZZ is far away from the other five sample groups and shows the largest differences in VOCs.

**Figure 4:**
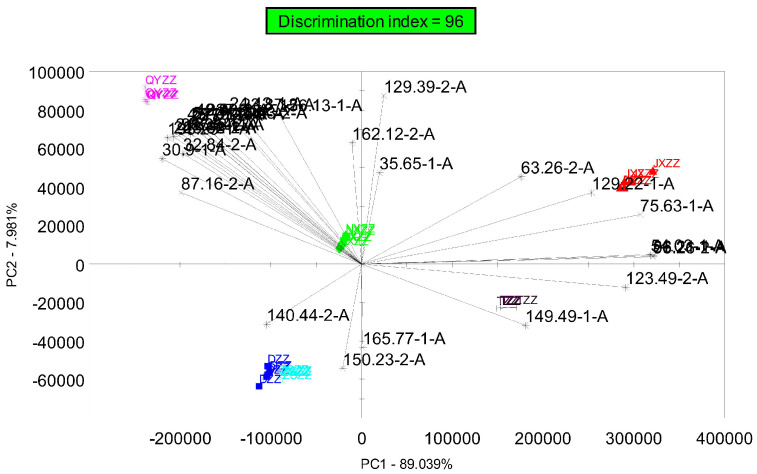
Principal component analysis and loading diagram of gardenia fruit samples.

The differences in VOCs between the different samples were further analyzed, and a loading diagram (Loading) was added on the basis of the principal component analysis (PCA) diagram, as shown in Figure 4. The factors in the Heracles loading diagram are chromatographic peaks. The chromatographic peaks in the original gas chromatogram of the samples were measured by using discrimination ability and peak area (discrimination ability > 0.900, peak area > 500), and the chromatographic peaks with an obvious discrimination, large peak area, and good separation effect were screened as the main contributing factors. The closer a factor is to the sample, the greater its contribution rate is. Comparing the loading diagram obtained after screening the chromatographic peaks with the PCA in Figure 3, it can be seen that the overall distribution trend of the samples in the two diagrams is consistent, indicating that the post-screening chromatographic peaks are representative and reflect the overall odor of the sample. The chromatographic peaks that make the main contribution are marked in the figure, and they were searched in the AroChemBase database (2021 version, Alpha MOS Corporation, Toulouse, France) according to the retention index of the chromatographic peaks. These chromatographic peaks were qualitatively analyzed, and information on the compounds causing the differences in the six gardenia fruit samples were obtained.

### 3.3. Compound Identification

Following the above screening, the retention times (Rts) of the chromatographic peaks that met the conditions for retention were converted into a retention index (RI) and qualitative analysis was performed in the AroChemBase database (2021 version, Alpha MOS Corporation, Toulouse, France) to obtain the compounds that potentially exist in each gardenia fruit sample and the properties of each sample. Sensory description information and qualitative results are shown in Table 1 and Table 2. In order to make the qualitative results of the chromatographic peaks more accurate, this study adopted a cross-qualification method by analyzing the retention index of two chromatographic columns, MXT-5 and MXT-1701, and then obtained the results from the database. The compound with the highest correlation coefficient among all the compounds retrieved was selected as the result display.

The odor threshold item in the table refers to the minimum concentration at which a specific odor can be perceived, indicating the compound odor strength in two media: air and water. The unit is mg/m3 in air and mg/kg in water. For two compounds with equal content in the same medium, the lower the threshold, the stronger the VOCs. As shown in Table 2, we calculated the average peak area of each compound, corresponding to the chromatographic peaks of gardenia fruit samples with different origins (the average is the mean of five parallel samples; MXT-5 and MXT-1701 were selected based on peak status). An FID detector, which is also a mass detector, was used to determine the peak area, representing the compound content. For different gardenia fruit samples corresponding to a compound, the larger the average peak area, the higher the compound content in that sample.

**Figure 5:**
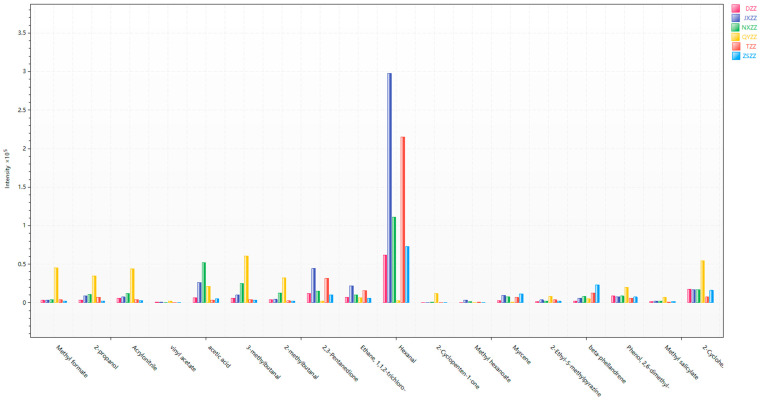
Histogram of differential compound contents.

A total of 18 VOCs were identified, as seen in Table 1. In order to compare the content differences of VOCs in gardenia fruit samples more intuitively, a histogram of the contents of differential compounds was drawn, with the VOCs as the abscissa and the average peak area as the ordinate, as shown in Figure 5. As seen from the histogram, the more obvious feature is QYZZ, which had the highest content of 11 compounds, including methyl formate, 2-propanol, acrylonitrile, vinyl acetate, acetic acid, and 3-methyl butanal. The contents of hexanal and myrcene in this sample were lower than in the other five samples. The content change in NXZZ is relatively stable, and its acetic acid content is higher than in other samples. The compound with the highest content in gardenia fruit is hexanal, among which the JXZZ sample had the highest content, followed by TZZ. The contents of 2,3-pentanedione, ethane, and 1,1,2-trichloro in JXZZ were higher than those in the other samples, and the contents of myrcene and beta-phellandrene in ZSZZ were higher than in other samples. The contents of 2,3-pentanedione, ethane, 1,1,2-trichloro, and hexanal in TZZ were relatively high, while the contents of other compounds were relatively low. DZZ did not show any significantly higher contents of components than the other gardenia fruit samples, and the contents of its compounds were generally lower than in other samples. The data in the Figure 5 show that the chemical composition of VOCs in gardenia fruit was relatively similar, but the same chemical composition of gardenia fruit from different sources has great differences, indicating differences in the quality of gardenia fruit from different sources.

### 3.4. Discriminant Factor Analysis (DFA)

**Figure 6:**
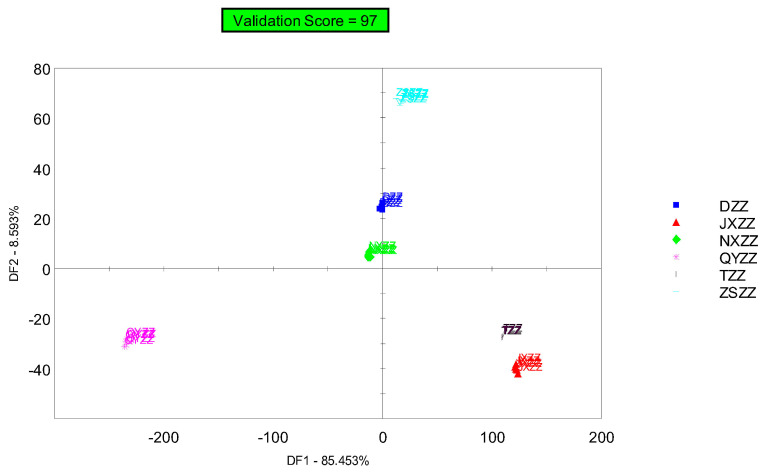
Discriminant factor analysis chart of different gardenia fruit samples.

Discriminant factor analysis (DFA) is a statistical analysis method that determines which category an individual belongs to [24]. It optimizes this distinction through the sample data of two or more known categories, maximizing the distance between groups while ensuring the smallest possible difference within the groups used for qualitative judgment. Based on principal component analysis (PCA), DFA can be performed to expand the differences between groups by reducing the differences within each group, thereby making gardenia fruit samples from different origins more distinguishable. The results are shown in Figure 6. As can be seen in the discriminant factor analysis chart of the gardenia fruit samples, DZZ and NXZZ are relatively close, TZZ and JXZZ are relatively close, and ZSZZ and DZZ are relatively close. QYZZ and the other five components are not located in the same quadrant, and the smell of the furthest component is significantly different. There is a difference between the DFA results and the PCA results in that the distance between DZZ and ZSZZ is closer, but the overall component differences are similar, which further validates the PCA results. The contribution of discriminant factor 1 (DF1) is 85.453%, the contribution of discriminant factor 2 (DF2) is 8.593%, and the cumulative contribution of the two is 94.046%, indicating that the DFA model can effectively distinguish different gardenia fruit samples, and that the discrimination effect is relatively good. The results obtained are the same as those of the PCA, which further validates the results of the PCA.

## 4. Discussion

China produces gardenia fruits in dozens of areas, and each region has its own characteristic varieties. Due to the influence of various factors, gardenia fruits from different production areas have distinct types and contents of chemical components, leading to differences in medicinal efficacy [25]. For the identification of gardenia fruit products from different sources, descriptions are mostly based on sensory attributes like appearance, color, aroma, and taste [26]. However, human olfactory perception is easily influenced by subjective factors. Electronic nose technology can specifically analyze chemical substances to provide a more detailed and objective scientific evaluation of gardenia fruit flavor. Comparing the chemical composition of gardenia fruits from different sources also provides an objective reference for identifying genuine products based on traditional experience [26], and more conducive to controlling gardenia fruit quality and reducing chaotic buying and selling.

In this experiment, the gas chromatography overlay of six batches of gardenia fruit samples shows that the sample with the most significant difference is QYZZ, followed by JXZZ. The difference between QYZZ and the other samples is reflected in all of the chromatographic peaks, which are generally higher than those of other samples, and the difference in JXZZ from the other samples is reflected in the individual chromatographic peak at 100s being higher than in the other samples. The PCA of gardenia samples shows that the positions of the DZZ, ZSZZ, NXZZ, and TZZ samples are relatively clustered in the figure and concentrated in the third and fourth quadrants, with JXZZ located in the first quadrant and relatively close to TZZ. QYZZ is located above the second quadrant and far away from other samples. The PCA results are consistent with the gas chromatograph. The graph analysis results are consistent. A total of 18 compounds were identified from the qualitative results of the differential chromatographic peaks. The main compound types identified were organic acid esters, ketones, aldehydes, and olefin. Combined with the differential compound content histogram, we can infer that acetic acid, 3-methyl butanal, 2,3-pentanedione, ethane, 1,1,2-trichloro, and hexanal are the difference markers of the six batches of gardenia fruit samples.

In the flavor characteristic evaluation, fruity, sour, sharp, and sweet were the main sensory results of the six batches of gardenia fruit samples. The compound with the greatest difference among the samples was hexanal, which exhibits fatty, fresh, fruity, and sweet flavors. Hexanal is a compound that naturally occurs in plants and it has a refreshing flavor similar to hay [27]. In the field of fruit seasoning, hexanal is often used to produce fruit flavors, and its content is higher in JXZZ and TZZ. Therefore, compared to other gardenias, JXZZ and TZZ are more suitable for fruit seasoning. Acetic acid presents acidic, sharp, and sour flavors, and it is best reflected in NXZZ. 2,3-Pentanedione has burnt, malty, and sweet flavors, and it has higher contents in JXZZ and TZZ. The content of 2-propanol in QYZZ is significantly higher than that in other gardenia fruits, indicating that QYZZ is more suitable for gardenia alcoholic beverages compared to other gardenia fruits as the only substance that produces an alcoholic odor. In addition, combining the flavor evaluation of VOCs in previous studies, it can be concluded that aldehyde compounds mostly present meaty and fatty aromas, and their odor thresholds are generally low [28], while ester compounds mostly express fruity aromas [29,30,31], which are often used in food additives and flavors, fragrances, and other fields. The flavor characteristics of ketone compounds are mainly fatty, creamy, and caramel [32]. 

Smell is an important criterion for evaluating food quality and distinguishing authenticity. With the development of science and technology, new bionic sensory technologies, such as electronic noses and electronic tongues, can be applied to quality control [33,34]. In terms of food safety, electronic noses can be used to detect pesticide residues or the maturity and freshness of fruits and vegetables, distinguish adulteration or authenticate edible oils and dairy products, and serve as smart packaging to perceive and detect food quality. Compared with the traditional evaluation methods of artificial nose smelling and oral taste, electronic noses have the advantages of good repeatability, electronic data, and easily understood description, and they can be used to conduct scientific and quantitative evaluations of smell and taste [35,36,37].

However, electronic noses also have their limitations, such as their discrimination ability not fully reflecting complex odor mixtures or high concentration gases, and matching with the AroChemBase database (2021 version, Alpha MOS Corporation, Toulouse, France) additionally has certain limitations [38]. In the subsequent detection of volatile substances in gardenia, improvements in sensor technology and updates to the AroChemBase database (2021 version, Alpha MOS Corporation, Toulouse, France), or the use of other instruments for detection, can make the qualitative and quantitative results of experiments more accurate, and achieve more improvements and applications in flavor evaluation.

At the same time, this study also has some shortcomings: there is only one sample from one region, and a single sample may not fully demonstrate the characteristics and diversity of the origin. In the future, larger sample sizes and more diverse regional studies will be conducted.

## 5. Conclusions

This study used Heracles Neo ultra-fast gas phase electronic nose technology to analyze the VOCs of six batches of gardenia fruit samples. A rapid detection method for gardenia fruit was established, and a total of 18 VOCs were identified and analyzed with PCA and DFA. The two models were verified, and obvious differences in VOCs were found between the gardenia fruits from different sources. This enabled the rapid identification of gardenia fruits from different sources based on odor information, establishing a rapid detection method for gardenia fruits. Among the six batches of gardenia fruit samples, QYZZ showed the greatest difference from the other samples, and its contents of methyl formate, 2-propanol, acrylonitrile, and other compounds were relatively high, while its content of hexanal was extremely low. The VOCs of JXZZ and TZZ were close, and the contents of hexanal and 2,3-pentanedione in JXZZ were higher. The VOCs of the DZZ, NXZZ, and ZSZZ samples were relatively close. The hexanal content in TZZ was higher, while the acetic acid content in NXZZ was higher. Acetic acid, 3-methyl butanal, 2,3-pentanedione, and hexanal may be important compounds responsible for the differences in VOCs of gardenia fruit from different sources. This study provides a feasible strategy for distinguishing gardenia fruits from different sources, making up for the shortcomings of traditional trait-based discrimination and providing reference values for the application of Heracles Neo ultra-fast gas phase electronic nose technology in the detection of other foods and drugs.

## Figures and Tables

**Table 1:** Differential chromatographic peak qualitative results and odor descriptions.

No	Compounds	CAS	RI (RT-5)	RI (RT-1701)	Odor Threshold (mg/m^3^)
1	Methyl formate	107-31-3	372	462	2.66 × 10³ (air)
2	2-propanol	67-63-0	478	603	90.00 (air)
3	Acrylonitrile	107-13-1	521	633	11.20 (air)
4	vinyl acetate	108-05-4	579	658	0.40 (air)
5	Acetic acid	64-19-7	619	-	0.60 (air)
6	3-methylbutanal	590-86-3	650	738	2.0 × 10^−^³ (air)
7	2-methylbutanal	96-17-3	661	743	3.3 × 10^−^³ (water)
8	2,3-Pentanedione	600-14-6	698	786	0.02 (air)
9	Ethane, 1,1,2-trichloro-	79-00-5	767	864	-
10	Hexanal	66-25-1	801	892	0.04 (air)
11	2-Cyclopenten-1-one	930-30-3	835	983	-
12	Methyl hexanoate	106-70-7	926	995	0.08 (water)
13	Myrcene	123-35-3	993	1023	0.04 (air)
14	2-Ethyl-5-methylpyrazine	13360-64-0	1014	1102	0.04 (air)
15	Beta-phellandrene	555-10-2	1033	1060	0.04 (water)
16	Phenol, 2,6-dimethyl-	576-26-1	1130	1292	2.0 × 10^−4^ (air)
17	Methyl salicylate	119-36-8	1210	1329	0.33 (air)
18	2-Cyclohexen-1-one, 2-methyl-5-(1-methylethenyl)-	99-49-0	1232	1383	0.50 (air)

**Table 2:** Average peak area of differential chromatographic peaks.

No	Compounds	CAS	Average Peak Area					
			DZZ	JXZZ	NXZZ	QYZZ	TZZ	ZSZZ
1	Methyl formate	107-31-3	3089	3197	3959	45,152	4168	2125
2	2-propanol	67-63-0	3327	8752	10,499	34,975	7142	2041
3	Acrylonitrile	107-13-1	6066	7430	12,022	44,024	4161	3000
4	vinyl acetate	108-05-4	955	670	426	2077	332	488
5	Acetic acid	64-19-7	6565	26,403	51,691	21,307	3516	5502
6	3-methylbutanal	590-86-3	6117	10,042	24,642	60,832	4079	3491
7	2-methylbutanal	96-17-3	3804	4438	12,837	32,102	2570	2256
8	2,3-Pentanedione	600-14-6	11,932	44,573	14,823	2115	31,796	10,405
9	Ethane, 1,1,2-trichloro-	79-00-5	6828	21,929	10,013	6634	15,761	5973
10	Hexanal	66-25-1	61,859	297,560	111,381	2550	215,125	72,907
11	2-Cyclopenten-1-one	930-30-3	594	440	1145	12,047	296	480
12	Methyl hexanoate	106-70-7	363	3567	1284	367	613	233
13	Myrcene	123-35-3	2461	9565	7882	816	6959	11,591
14	2-Ethyl-5-methylpyrazine	13360-64-0	1627	3755	2304	8534	3792	1896
15	Beta-phellandrene	555-10-2	2354	5797	8098	5104	12,887	23245
16	Phenol, 2,6-dimethyl-	576-26-1	8954	7733	8669	20,162	6015	7454
17	Methyl salicylate	119-36-8	1598	2342	1953	7365	703	1528
18	2-Cyclohexen-1-one, 2-methyl-5-(1-methylethenyl)-	99-49-0	17,808	17,036	16,982	54,251	7442	16,010

## Data Availability

The original contributions presented in the study are included in the article, further inquiries can be directed to the corresponding author.
